# Integrated Life Skills Training and Executive Function Strategies in Children With Autism Spectrum Disorder in Qatar: A Pilot Study of a Randomized Controlled Trial

**DOI:** 10.7759/cureus.52809

**Published:** 2024-01-23

**Authors:** Bara M Yousef, Naresh Bhaskar Raj, Wan-Arfah Nadiah, Brightlin N Dhas, Ahmad M Mansour, Samah A Abd Alhadi, Florence V Rosal, Marnel M Dizon

**Affiliations:** 1 Rehabilitation, Hamad Medical Corporation, Doha, QAT; 2 Faculty of Health Sciences, University Sultan Zainal Abidin, Kuala Nerus, MYS

**Keywords:** effectiveness, occupational therapy, visual motor integration, sensory integration/processing, daily life skills, executive function strategies, autism spectrum disorder

## Abstract

Background and aim

Executive function (EF) impairment is common in children with autism spectrum disorder (ASD). EF strategies are considered effective in improving the therapeutic outcomes of children with ASD. This study primarily aimed to explore whether integrating EF strategies combined with regular occupational therapy intervention is more effective in improving daily life skills (DLS) and sensory integration/processing (SI/SP) skills than regular occupational therapy alone in children with ASD and secondarily aims to assess treatment outcomes on improving visual motor integration (VMI) skills.

Methods

A total of 17 participants (13 males, mean age 4.29 years, standard deviation 0.66) completed the study. Following the baseline assessments, the participants were randomly assigned to the treatment group (45-minute once-weekly individual occupational therapy plus EF strategies) or control group (45-minute once-weekly individual therapy sessions alone). All participants received one intervention per week for 14 weeks. All children were systematically evaluated using a pediatric functional independent measure (WeeFIM) and the Verbal Behavior Milestones Assessment and Placement Program (VB-MAPP) to assess DLS, the Short Sensory Profile 2 (SSP2) to assess SP/SI, and the Beery VMI test (Beery VMI) to assess VMI. Assessments were conducted at baseline, seven weeks, and 14 weeks of treatment.

Results

The analysis of the results between the treatment and control groups revealed that the treatment group had greater gains and significant differences in the mean values of both the WeeFIM and SSP2. In addition, notable distinctions were observed in the VB-MAPP transition subscale; although these differences did not reach statistical significance, they were clinically significant. Minimal differences were noted in the VMI between the two groups. Nevertheless, both groups showed statistically significant improvements across all outcome measures.

Conclusions

Our study provides preliminary evidence of the efficacy of EF strategies combined with regular occupational therapy for DLS, SP/SI, and VMI in children with ASD. The differences between the groups support further evaluation of the effectiveness of EF strategies for the next stage of a larger randomized clinical trial.

## Introduction

Autism spectrum disorder (ASD) is a neurodevelopmental disorder characterized by socio-communicative impairments and restricted, repetitive patterns of behaviors and interests [1].

The prevalence of ASD is increasing globally, with an estimated median prevalence of 65/10,000 [2]. The Centre for Disease Control and Prevention reported that approximately one in 36 children eight years of age in the USA was estimated to have ASD [3].

Children with ASD may have other comorbidities, including epilepsy, sleep disorders, gastrointestinal disorders, toileting problems, behavioral disorders, learning disorders, attention deficit hyperactivity disorder, intellectual disability, anxiety, and depression [4]. Deficits in executive function (EF), including working memory, inhibitory control, self-regulation, and cognitive flexibility, which involve planning and problem-solving, were also reported among children with ASD. These children require treatment programs that involve a multidisciplinary team targeting functional and life domains. Impairments in EF skills will subsequently impact participation in these domains and affect the quality of life and participation [5]. Therefore, developing effective intervention strategies that incorporate EF skills is crucial. To achieve this, we need to understand the nature of EF impairment in children with ASD and the reported treatment recommendation that aligns with the occupational therapy (OT) program.

EF impairment in children with ASD

More than half of children with ASD have impairments in EF skills despite their intelligence quotient (IQ) being within the normal range or higher [6]. Compared to typically developing children, children with ASD were reported to have impairments in all EF domains, such as inhibitory control, self-regulation, planning, and problem-solving [7]. Impairment in EF skills could affect core ASD symptoms such as stereotypic and repetitive behaviors, thereby influencing the ASD phenotype [8]. For example, inhibitory control and self-regulation were found to be highly correlated with the presence of stereotypic and repetitive behaviors in children with ASD. Other EF skills, such as working memory, shifting, and inhibition, are suggested to be associated with other areas of core ASD symptoms, such as social communication [9]. Consequently, children with ASD perform poorly on verbal working memory tasks compared to those with typical language development [10].

EF training for children with ASD

Over the last decade, several studies have investigated the effect of EF training on children with ASD. Most studies reported improvement in core executive skills, like problem-solving abilities, as measured via direct assessments [11]. Improvements in planning and organization skills were reported in another study using the Unstuck and On Target intervention that targets flexibility, planning, and problem-solving compared to the social skill intervention [12].

Some studies have also discussed self-regulation, a key EF skill in children with ASD. Improvements have been observed in participants' ability to regulate behavior and use cognitive resources after implementing EF training and a social competence intervention [13]. Likewise, only a few studies have investigated the outcomes in social communication and language skills following EF training, and those researchers suggested that EF interventions are associated with improvements in social communication skills and better outcomes in language skills in children with ASD [14].

Improvements in social communication skills have also been reported in 5-7-year-old children with ASD following the use of EF strategies in schools, and positive results were observed in classroom engagement, social interaction, adaptive communication, and social skills [15]. Computerized EF training can also be a useful technique to improve EF skills among children with ASD of different ages when implemented by teachers in regular classrooms [16]. However, computerized EF training was shown to be more beneficial for older children aged 8-12 years than those aged 4-5 years. Conversely, non-computerized training has shown better results because it involves more in-person, trainer-trainee interactions than computerized cognitive training [17].

Most of the published studies on EF training have examined its effectiveness in improving social communication skills when implemented as a stand-alone strategy or combined with social communication training [11]. No study has explored the outcomes of EF training in improving daily life skills (DLS) or sensory integration/sensory processing (SI/SP) skills [18].

Aim of the current study

This study aimed to 1) assess and compare the effects of combining EF strategies with DLS and SI/SP training versus using DLS and SI/SP training independently in enhancing self-care, SP/SI, and visual motor integration (VMI) skills in children with ASD; 2) to investigate the impact of DLS and SI training on improving self-care, SP/SI, and VMI skills in children with ASD; and 3) to examine the outcomes of integrating EF strategies with DLS and SI training. We hypothesize that in children with ASD, EF training combined with DLS and SI/SP training is more effective in improving self-care and SI skills than DLS and SI/SP training alone.

## Materials and methods

Study design

This study protocol was designed in line with other previous protocols and studies [13,15,17] that demonstrated that EF training for children with ASD can be effective and that SI/SP training and DLS training for children with ASD are also effective; however, our study goes a step further by combining EF training with SI/SP and DLS training.

The current study was a single-blinded, randomized control trial (RCT) with two arms: OT+EF (treatment) versus OT (control). The duration of intervention and length of each session were the same for both groups in a 45-minute session/week for 14 weeks. The two groups were compared at baseline, seven, and 14 weeks of treatment. Informed consent was obtained from the parents of participants. 

Participants

Children with a confirmed diagnosis of ASD were referred to the Autism Program at Almaha Center, HMC. ASD diagnosis was confirmed by a multidisciplinary team at the Child Development Center at HMC. The diagnostic team included consultant neurodevelopmental pediatricians, occupational therapists, speech therapists, and psychologists. All of them specialized in ASD diagnosis. Children included in the study had a confirmed diagnosis of ASD, were referred to the autism program, and were between the ages of three and five years. All eligible children, irrespective of their native languages, were included. Exclusion criteria were children with severe impairments in learning and linguistic acquisition skills with a score of four on any item of Barrier Assessment on the Verbal Behavior Milestones Assessment and Placement Program (VB-MAPP) and whose parents were unwilling to give consent. Children who did not complete 14 intervention sessions or who did not complete all outcome measures and those who underwent other interventions that may affect the study results were considered dropouts and hence excluded from the analysis. Study screening was performed by the primary investigator (PI) after the parents provided written informed consent. Parents were asked to come for another session during which the PI performed screening based on the eligibility criteria. All eligible children whose parents signed the consent form were enrolled in the study. The PI explained the study enrollment for parents on the same day of screening.

Sample size determination

A priori power analysis was conducted using G*Power 3.1.9.6 (Heinrich-Heine-Universität Düsseldorf, Germany) considering an effect size of 0.8 and a 5% margin of error, power of 0.95 based on previous studies [13,15,16]. The results of the power analysis indicated that 84 participants are required to detect group differences, and given the likelihood of a 10% dropout rate during the study, we planned to recruit 92 participants in the originally planned large RCT to accommodate potential attrition. In this pilot study, 23 participants were enrolled.

Randomization and blinding

Participants were randomized (1:1) and assigned to the treatment or the control group using a computer-generated sequence. Baseline and outcome assessments were performed by independent evaluators blinded to the study groups. The evaluators are trained to conduct the study assessments.

Ethical considerations

This study was approved by the Institutional Review Board of Hamad Medical Corporation (HMC; IRB-HMC-2021-011) and the Medical Research Center (MRC-01-22-509), and ethics committee approval was also received for this study from Universiti Sultan Zainal Abidin (UniSZA) Human Research Ethics Committee (UHREC; study code UniSZA/UHREC/2022/443). Parents of children who were admitted to the Autism Program were asked whether they agreed to join the study or not; if they agreed, they were asked to sign the consent form, which was given to them by the PI. There are no major risks associated with this study; however, children may get upset while performing the tasks. All data collection forms were approved and stamped by the Medical Research Center (MRC) and Institution Review Board (IRB) at the Hamad Medical Corporation (HMC), Qatar. Parents of all children referred to the Autism Program were informed about the research, and those who agreed to participate were screened by the PI based on the eligibility criteria. Potential participants were given the choice of whether to participate in the study, and additional time was provided if required to make the decision. Informed consent was obtained from those parents whose children were eligible for the study. The participant names were replaced by a code number in the dataset for anonymity. All data files (paper forms) have been stored securely in safe or locked file cabinets inside a secured room, electronic forms have been saved securely only on HMC computers and networks, and the patient data file has been encrypted with a password. The study protocol was previously posted to the Research Square preprint server on September 06, 2023.

Study measures

An overview of all outcome measures used and timing is shown in Table 1.

**Table 1:** Study schedule for recruitment, interventions, and outcome measurements VB-MAPP - Verbal Behavior Milestones Assessment and Placement Program; SSP2 - Short Sensory Profile 2; WeeFIM - Pediatric Functional Independent Measure; VMI - visual motor integration

Study period	Enrollment	Allocation	Post-allocation	
Timepoint (week)	-1	0	7	14
Informed consent	X			
Eligibility (screening)	X			
Randomization	X			
Assessment				
VB-MAPP	X	X	X	X
SSP2		X	X	X
WeeFIM		X	X	X
VMI		X	X	X

Screening Measures

The Verbal Behavior Milestones Assessment and Placement Program (VB-MAPP) was used to determine eligibility. VB-MAPP has five dimensions: milestones assessment and barriers assessment, which were used for inclusion criteria; transition assessment; task analysis skills tracking; and placement and individualized education program goals. VB-MAPP barrier assessment uses a five-point scale (0-4), with 0 being the lowest and 4 being the highest score. VB-MAPP is the most widely used instrument for curriculum development and treatment planning in the field of ASD training. Moreover, it also has good psychometric properties and is a reliable and valid measure [19].

Primary Outcome Measures

VB-MAPP transition assessment was used to measure improvements in self-help skills. VB-MAPP transition assessment uses a four-point scale (1-4), with 1 being the lowest and 4 being the highest score. VB-MAPP transition assessment includes good psychometric properties and is a reliable and valid measure [19].

The Pediatric Functional Independent Measure™ (WeeFIM) was used to measure improvements in self-care and social cognition skills. It consists of 18 items with three domains: self-care, mobility, and social cognition. Each item uses a seven-level method of 1-7 points. In this study, only self-care and social cognition domains were used. WeeFIM is shown to demonstrate high internal consistency responsive to change and good convergent validity [20].

The Short Sensory Profile™ 2 (SSP2) was used to measure improvement in sensory processing. SSP2 comprises a 34-item caregiver questionnaire, and all the items are scored on a five-point Likert scale (ranging from always to never). A lower score indicates the presence of more atypical sensory responses. SSP2 has a total score and seven subsection scores, which can be used to classify children's sensory impairments into four categories: seeking, avoidance, sensitivity, and registration. SSP2 is one of the most commonly used measures of sensory features in children with ASD, and responsiveness to change, concurrent validity, and reliability appear satisfactory [21].

Secondary Outcome Measure

The Beery Visual Motor Integration Test (VMI) was used to measure changes in visual motor integration. VMI contains 30 items and uses a binomial scale with 0 denoting the absence and 1 denoting the presence of skill. VMI is a widely used visual motor assessment tool because of its extensive and well-documented psychometric properties. Its inter-rater reliability ranges from 0.92-0.98, and it has a test-retest reliability correlation of 0.92 for a two-week interval. Based on the norming studies [22], VMI is responsive to change and does not exhibit cultural bias [23].

Intervention

A 14-week occupational therapy training program was conducted. One session was run each week to form 14 sessions over 14 consecutive weeks. The duration of each session was 45 min. The program consisted of EF strategies and training, DLS training, and SI/SP training.

EF strategies and training were conducted by a PI and a trained therapist only for the treatment group. EF strategies were implemented during the DLS, and SI training targeted the following areas: using a monitoring system for each child, improving the sense of time, externalizing important information, externalizing motivation, and personalizing the command and praise along with direct intervention at the point of performance. To ensure the standardization of EF training and strategies, each clinician received a customized kit curated by the PI based on the current evidence of EF training for children with neurodevelopmental disorders. Each kit came with a set of guidelines that detailed how to use EF strategies. A kid-friendly monitoring board with pictures attached using Velcro for all the activities done during sessions was also present. Beside each picture, a spot for therapists to evaluate how well the child did, using Velcro symbols for right or wrong answers, was observed. This setup allowed for flexibility in adjusting the pictures and symbols based on the tasks and the child's individual progress. The board also included sequential numbers for the tasks.

At the bottom of the board, a designated area where therapists could place a picture of a reinforcement or reward to be given to the child upon completion of the required tasks was present where the board was kept in front of the child and within the child's visual field.

Another essential component of the kit included digital and sand timers (two, three, and five minutes) to enhance the child's sense of time and promote adherence to the designated time frames for each task and engagement. Therapists were guided to prioritize productivity initially and then accuracy later, especially in the early stages of training. Therapists were encouraged to reward the child for task completion, irrespective of accuracy, and to gradually increase the complexity and difficulty of the tasks. Chaining techniques, particularly for activities related to DLS training, were recommended, and to maintain motivation and accommodate fidgeting, therapists have also been guided to alternate between tabletop activities, DLS tasks, and sensory activities.

In addition, each kit included various EF training activities with varying levels of difficulty. These activities encompassed such tasks as navigating simple mazes, engaging in sequencing and categorization exercises that require planning and problem-solving skills, and fostering emotional perception and self-regulation.

Children in the treatment and control groups received DLS training and SI/SP training. The DLS training specifically targeted essential daily activities such as grooming, feeding, dressing and undressing, toileting, and community reintegration. This training was designed to enhance VMI skills in children, incorporating various techniques for activities of daily living, including prompting, shaping, forward and backward chaining, and modeling. The training was tailored to the individual level of independence determined by the WeeFIM score. The curriculum used for self-care training was based on VB-MAPP, and we used various tools, such as dressing aids, simulated feeding activities, and grooming aids. Visual support was also integrated to assist in self-care tasks.

The intervention featured the implementation of sensory therapies, such as deep pressure, brushing, weighted vests, and swinging. Tailored treatment activities were designed for individual needs, incorporating a carpeted scooter board. In one activity, the child lay in a prone position on the scooter board to ascend a ramp, followed by turning the scooter board around to descend the ramp and land in a cushioned area with mats and pillows featuring various textures [18,24,25]. This intervention, which was grounded in play with the active involvement of the child, took place in a spacious gym equipped with mats and a variety of suspended swings, large balls, a climbing wall, carpeted barrels, large inner tubes, and foam blocks. These elements provided opportunities for engaging in active, guided sensory-motor play. In addition, we conducted sensory interventions in a dedicated sensory room, which featured interactive soundboards, interactive light fiber optics, a bubble tube projector, and other devices aimed at providing multisensory stimulation and training [26,27].

## Results

**Figure 1:**
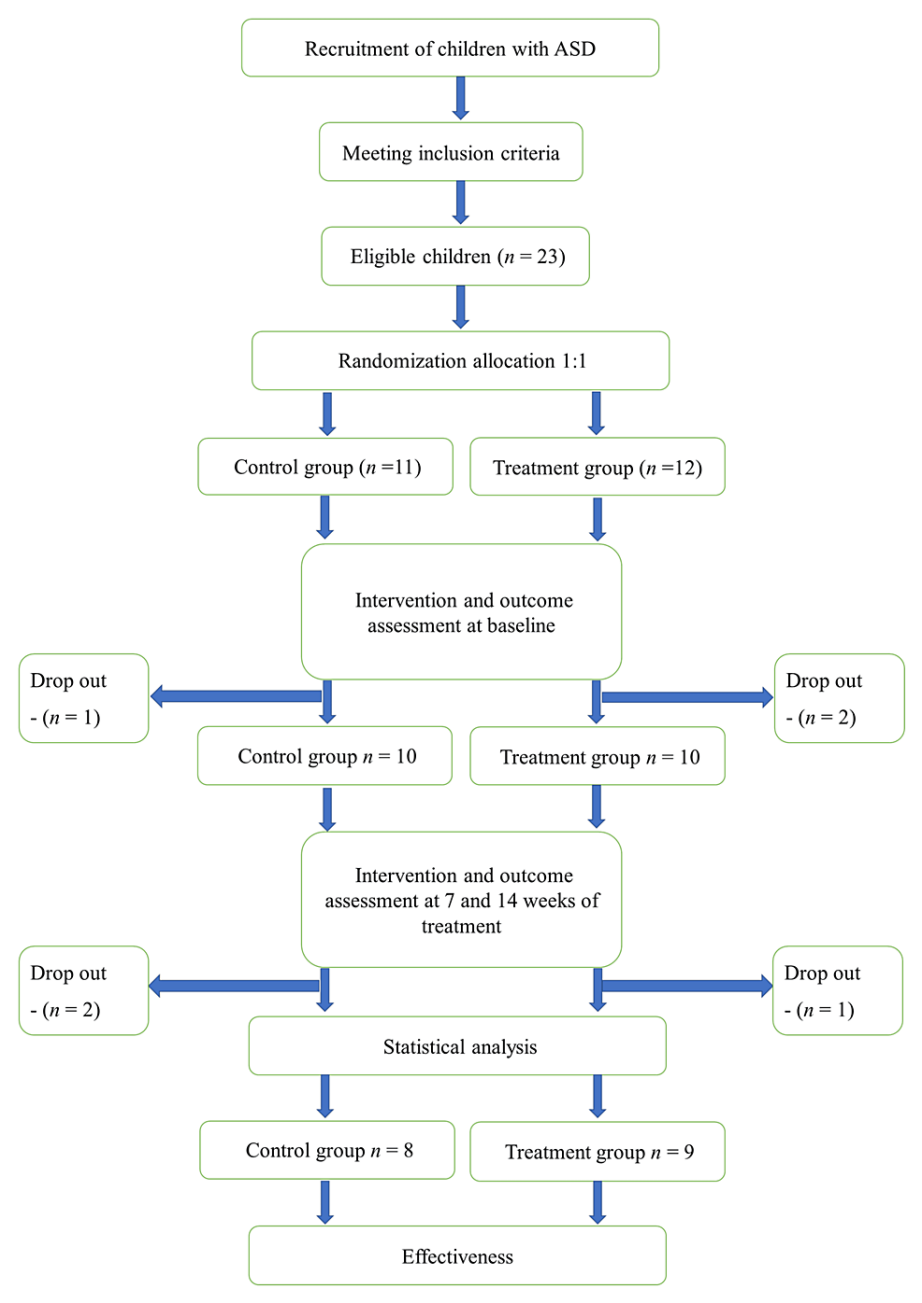
Study schedule for recruitment, interventions, and outcome measurements ASD - autism spectrum disorder

Twenty-three participants were enrolled in this study. One participant in the treatment group and two participants in the control group dropped out after the baseline assessment. Another three participants dropped out after the seven-week assessment and did not complete the required reassessments and interventions owing to unexpected family events, transportation, work responsibilities, childcare commitments, and no-shows. Those participants who dropped out after seven weeks were included in the descriptive purpose data analysis, whereas 17 participants who completed all training sessions were included in the comparative data analysis. There were nine participants in the treatment group and eight participants in the control group. Figure 1 illustrates the flow of randomized participants.

The baseline demographics and clinical characteristics of the 20 participants did not differ significantly between the observed groups (all p>0.05). Table 2 lists the participants' demographic and clinical characteristics.

**Table 2:** Demographic of the study participants M - male; F - female

Demographic characteristic	Treatment group	Control group	p-value
Age, years (m±SD)	4.5±0.68	4.1±0.53	0.159
Gender (M/F)	9/1	7/3	0.582

The primary outcome measures used in this study were the VB-MAPP, SP2, and WeeFIM. The secondary outcome was VMI. Normally distributed data and results were presented as mean and standard deviation (SD), whereas the median and interquartile range (IQR) were used for skewed/ordinal data. Categorical data were summarized using frequencies and percentages. We assessed the associations between two or more categorical variables using the Pearson chi-square and/or Fisher exact tests as appropriate. An unpaired test was used to compare the mean scores between the two independent groups. Changes in the pre and post-test scores measured at various time points within and between groups were compared using a linear-mixed regression model and analysis of covariance. A two-sided p-value <0.05 was considered statistically significant, and all key results were presented with their corresponding 95% confidence interval. We performed all statistical analyses using SPSS version 28.0 (IBM Corp., Armonk, NY) [28].

Table 3 summarizes the statistical analysis of the inferential statistics for the within- and between-group comparison of the clinical and kinematic outcomes of the study's dependent variables at baseline, seven-week reassessment, and 14-week reassessment.

**Table 3:** Distribution of outcome measurements at three time points Week 0: outcome measures at baseline; week 7: after the seven-week treatment period; week 14: after 14-weeks VB-MAPP - Verbal Behavior Milestones Assessment and Placement Program; SSP2 - Short Sensory Profile 2; WeeFIM - Pediatric Functional Independent Measure; VMI - visual motor integration

Dependent variables	Week 0				Week 7				Week 14			
	Treatment group, n=9		Control group, n=8		Treatment group, n=9		Control group, n=8		Treatment group, n=9		Control group, n=8	
	Mean (SD)	Min-Max	Mean (SD)	Min-Max	Mean (SD)	Min-Max	Mean (SD)	Min-Max	Mean (SD)	Min-Max	Mean (SD)	Min-Max
WeeFIM	15.4 (4.6)	10-23	15.3 (5.78)	9-24	19 (3.6)	13-24	17.75 (6.27)	11-26	23.89 (6.13)	16-33	20.25 (6.38)	12-30
SSP2	95.8 (6.80)	53-152	87.8 (19.83)	55-115	81.2 (22.33)	45-116	82.6 (22.02)	51-117	67.4 (15.67)	40-92	75.5 (21.22)	48-110
VB-MAPP	7.6 (3.27)	4-13	8 (3.505)	3-14	9.3 (3.31)	4-15	8.7 (3.10)	4-13	10.6 (2.73)	7-15	8.8 (2.69)	4-13
VMI	4.5 (3.20)	0-11	5.8 (4.32)	0-13	9.2 (3.34)	3-14	9.3 (3.88)	3-15	11.3 (2.73)	8-16	11.2 (2.25)	8-14

**Figure 2:**
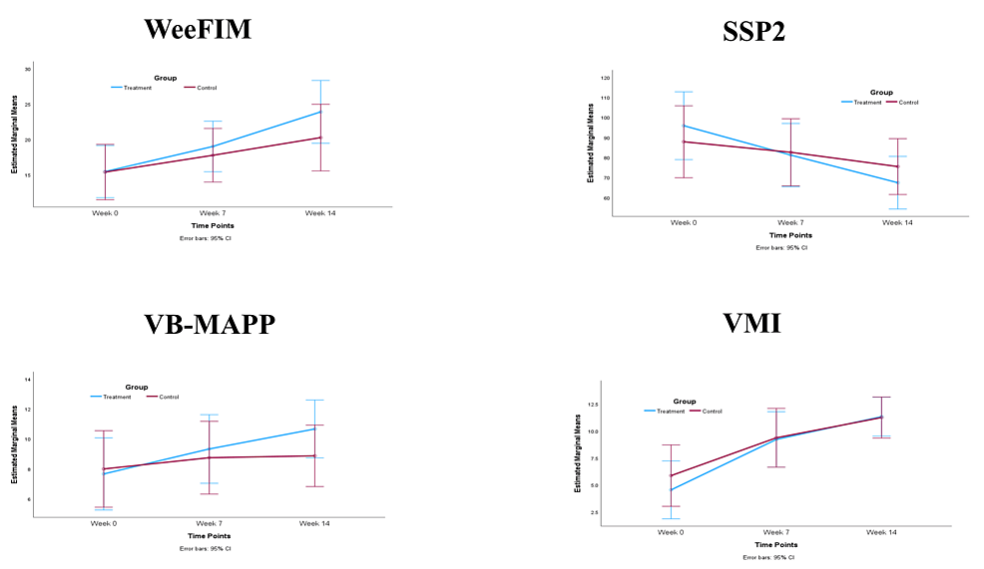
Changes in the mean values of the WeeFIM, SSP2, VB-MAPP, and VMI within the treatment and control groups Week 0: outcome measures at baseline; week 7: after the seven-week treatment period; week 14: after 14-weeks VB-MAPP - Verbal Behavior Milestones Assessment and Placement Program; SSP2 - Short Sensory Profile 2; WeeFIM - Pediatric Functional Independent Measure; VMI - visual motor integration

For the within-group comparison, we found significant improvements within each group when comparing the baseline with subsequent time points. For the WeeFIM, SSP2, and VMI, all p-values were <0.0001, with effect sizes for the treatment group of 1.56, 2.35, and 2.28 and for the control group of 0.813, 0.598, and 1.56, respectively. For VP-MAP, the p-value was 0.0001, with an effect size for the treatment group of 1.00 and for the control group of 0.256. In the between-group comparison, the treatment group exhibited a noticeable and clinically significant meaningful difference in the mean magnitude on the WeeFIM and VB-MAPP at the seven and 14-week time points and on the SSP2 at the 14-week point. This difference tended to increase over time. We found no differences in VMI between the groups. In addition, the differences in all outcome measures did not reach statistical significance (p>0.05 for all), and the effect sizes ranged from 0.4 to 0.7. Figure 2 illustrates the mean within-group changes in the WeeFIM, SSP2, VB-MAPP, and VMI in the treatment and control groups. Table 4 shows the results of the repeated-measures analysis, including baseline, week seven, and week 14 assessments.

**Table 4:** Statistical analysis of repeated measures of the dependent variables in the four study assessments *Significant difference between improvement in the study groups (p<0.001). Cohen's d uses the pooled standard deviation. VB-MAPP - Verbal Behavior Milestones Assessment and Placement Program; SSP2 - Short Sensory Profile 2; WeeFIM - Pediatric Functional Independent Measure; VMI - visual motor integration

Dependent variables	Between-group		Within-group		
	p-value	Effect size, d	p-value	Effect size, d	
				Treatment group	Control group
WeeFIM	0.526	0.582	<0.0001^*^	1.56	0.813
SSP2	0.962	0.436	<0.0001*	2.35	0.598
VB-MAPP	0.640	0.659	0.001	1.00	0.256
VMI	0.749	0.033	<0.0001*	2.28	1.567

Our study thus provides preliminary evidence for the efficacy of EF strategies combined with a regular occupational therapy intervention on DLS, SP/SI, and VMI in children with ASD, thereby supporting further evaluation of the effectiveness of EF strategies in the context of a larger randomized clinical trial.

## Discussion

Children with ASD present impairment with several EF skills with different levels of impairment. EF strategies and training are important components of the treatment program for children with ASD [12,16]. The EF strategies implemented in the present study share similarities with those employed in other studies focusing on EF strategies, such as externalizing important information [29] and incorporating external reinforcement and motivation within the intervention [30]. The implemented strategies, along with EF training [12], aim to address various EF skills such as working memory, inhibitory control, planning, and problem-solving [13-15]. This study is situated within individual OT sessions in an outpatient hospital setting, differing from earlier studies that were conducted in classroom settings. Furthermore, this study diverges by not using assessments specifically focusing on social skills and self-regulation [11,12].

In comparison to earlier studies primarily exploring the impact of EF training on enhancing social skills and specific EF skills [12-14], this study uniquely evaluates the outcomes of EF training on enhancing DLS and SI/SP [18]. Although the frequency of intervention sessions varied across studies [13,15,24-27], the 14-session duration in this study aligns with the current practice in pediatric OT, where definitive guidelines for intensity and frequency of treatment are currently lacking. The positive outcomes observed underscore the need for further investigation into the optimal frequency and intensity of intervention. Consistent with our hypothesis, we found greater improvement in overall DLS and SP/SI skills in the treatment group than in the control group. We also found a noticeable and clinically significant meaningful difference in the mean magnitude between the groups at the seven and 14-week time points. Although it did not reach statistical significance, this difference tended to increase over time, and improvements in post-test are attributed to the effect of the interventions themselves. This finding is consistent with previous work showing that intervention to improve EF has positive outcomes [13,14,16-19] or, in any case, is correlated with them. Contrary to our hypothesis, the VMI skills improved to a similar extent between the two groups, although there was a statistically significant improvement within both groups in all outcome measures and high effect size. The results of this study support the assumption that this intervention has a positive effect.

The information on the preparation period of this study can be of great interest to other studies for designing and performing relevant studies. The findings of this study might give rise to further research; moreover, investigations on other factors, such as the severity of ASD, SI/SP, and DLS training techniques and parent's compliance with treatment and educational level, might be conducted in the future. The outcome of the study will also contribute to strengthening the limited existing evidence on the efficacy of EF enhancement strategies for children with ASD. The program can also be integrated into the service delivery mode of an occupational therapy program in different healthcare centers for children with ASD in Qatar; however, the authors would like to acknowledge some limitations in this section.

This study has some limitations that must be considered. First, no follow-up assessments were conducted after the children were discharged. It would be beneficial for studies to evaluate whether the effects of EF strategies are sustained during the follow-up period. Second, a degree of individualization in applying EF strategies for participants was observed. Although we standardized the duration, frequency, and categories of the training, investigating whether the specific techniques used in EF strategies influence treatment outcomes would be intriguing for future studies. Lastly, although we observed improvements in the study outcomes between the groups, these improvements did not reach significance, possibly owing to the sample size of this pilot study. Future studies could consider recruiting a larger number of participants to determine if a larger sample size yields statistically significant results.

## Conclusions

This investigation is among the pioneering studies focusing on the usage of EF strategies for children diagnosed with ASD in Qatar. The primary aim of this study was to assess the efficacy of incorporating EF strategies into occupational therapy programs designed for young children with ASD. This research, conducted as a pilot study of a single-blind randomized controlled pilot study, furnishes empirical evidence supporting the effectiveness of EF strategies when integrated with standard occupational therapy programs. The results of the trial advocate for the inclusion of EF strategies in multidisciplinary programs tailored for children with ASD, offering a potential alternative approach to delivering occupational therapy services in local healthcare facilities, not only in Qatar but also in other countries. However, the observed effects of the training were modest, prompting the need for further exploration in large clinical trials.
